# Feedback stabilization of probabilistic finite state machines based on deep Q-network

**DOI:** 10.3389/fncom.2024.1385047

**Published:** 2024-05-02

**Authors:** Hui Tian, Xin Su, Yanfang Hou

**Affiliations:** ^1^Key Laboratory of Industrial Internet of Things and Networked Control, Ministry of Education, Chongqing University of Posts and Telecommunications, Chongqing, China; ^2^School of Electrical and Electronic Engineering, Chongqing University of Technology, Chongqing, China

**Keywords:** probabilistic finite state machine (PFSM), deep Q-network (DQN), feedback stabilization, artificial neural network (ANN), controller

## Abstract

**Background:**

As an important mathematical model, the finite state machine (FSM) has been used in many fields, such as manufacturing system, health care, and so on. This paper analyzes the current development status of FSMs. It is pointed out that the traditional methods are often inconvenient for analysis and design, or encounter high computational complexity problems when studying FSMs.

**Method:**

The deep Q-network (DQN) technique, which is a model-free optimization method, is introduced to solve the stabilization problem of probabilistic finite state machines (PFSMs). In order to better understand the technique, some preliminaries, including Markov decision process, ϵ-greedy strategy, DQN, and so on, are recalled.

**Results:**

First, a necessary and sufficient stabilizability condition for PFSMs is derived. Next, the feedback stabilization problem of PFSMs is transformed into an optimization problem. Finally, by using the stabilizability condition and deep Q-network, an algorithm for solving the optimization problem (equivalently, computing a state feedback stabilizer) is provided.

**Discussion:**

Compared with the traditional Q learning, DQN avoids the limited capacity problem. So our method can deal with high-dimensional complex systems efficiently. The effectiveness of our method is further demonstrated through an illustrative example.

## 1 Introduction

The finite state machine (FSM), also known as finite automata (Yan et al., 2015b), is an important mathematical model, which has been used in many different fields, such as manufacturing system (Wang et al., 2017; Piccinini et al., 2018), health care (Shah et al., 2017; Zhang, 2018; Fadhil et al., 2019), and so on. The deterministic finite state machine (DFSM) is known for its deterministic behaviors, in which each subsequent state is uniquely determined by its input event and preceding state (Vayadande et al., 2022). However, DFSMs may not be effective in dealing with random behaviors (Ratsaby, 2019), for example, the randomness caused by component failures in sequential circuits (El-Maleh and Al-Qahtani, 2014). To address the challenge, a probabilistic finite state machine (PFSM) was proposed in the study by Vidal et al. (2005), which provides a more flexible framework for those systems that exhibit random behaviors. Especially, it gives an effective solution to practical issues, such as the reliability assessment of sequential circuits (Li and Tan, 2019). Therefore, the PFSM offers a new perspective for the theoretical research of FSMs.

On the other hand, the stabilization of systems is an important and fundamental research topic, and there have been many excellent research results in various fields, for example, Boolean control network (Tian et al., 2017; Tian and Hou, 2019), time-delay systems (Tian and Wang, 2020), neural networks (Ding et al., 2019), and so on. The stabilization research of FSMs is no exception and has also attracted the attention of many scholars. The concepts of stability and stabilization of discrete event systems described by FSMs were given in the study by Özveren et al. (1991). A polynomial solution of stability detection and a method for constructing stabilizers were presented. Passino et al. (1994) utilzed the Lyapunov method to study the stability and stabilization of FSMs. Tarraf et al. (2008) proposed some new concepts, including gain stability, incremental stability and external stability, and then established a research framework for robust stability of FSMs. Kobayashi et al. developed a linear state equation representation method for modeling DFSMs in the study by Kobayashi (2006) and Kobayashi and Imura (2007) and derived a necessary and sufficient condition for DFSM to be stabilizable at a target equilibrium node in the study by Kobayashi et al. (2011).

However, as we know, the FSM is most often non-linear. Moreover, none of the above methods are convenient when analyzing and designing various FSMs. In the last decade, scholars applied the semi-tensor product (STP) of matrices to FSMs and derived many excellant results. First, with the help of STP, an algebraic form of DFSMs was given in the study by Xu et al. (2013). This algebraic form is a discrete-time bilinear equation. Then, the classic control theory can be used to investigate FSMs. Especially, under the algebraic form, necessary and sufficient conditions for the stabilizability of DFSMs were derived in the study by Xu et al. (2013), and a state feedback controller was obtained by computing a corresponding matrix inequality. Moreover, Yan et al. (2015a) provided a necessary and sufficient condition to check whether a set of states can be stabilized. Han and Chen (2018) considered the set stabilization of DFSMs and provided an optimal design approach for stabilizing controllers. Later, Zhang et al. used the STP method to investigate PFSMs and non-deterministic FSMs. Specifically, a necessary and sufficient condition for stabilization with probability one and a design method for optimal state feedback controller were provided in the study by Zhang et al. (2020a). Moreover, a systematic procedure was designed to get a static output feedback stabilizer for non-deterministic FSMs in the study by Zhang et al. (2020b). Although the STP method is very useful in analyzing discrete event systems, including various FSMs, it suffers from high computational complexity and can only handle small-scale or even micro-scale discrete event systems. To solve the problem, this study refers to techniques developed by Acernese et al. (2019) to solve the stabilization problem of high-dimensional PFSMs, and then provides a reinforcement learning algorithm to compute a state feedback stabilizer for PFSMs. The algorithm is especially advantageous in dealing with high-dimensional systems.

The rest of this study is arranged as follows: Section 2 introduces some preliminary knowledge, including PFSM, Markov decision process (MDP), deep Q newtwork (DQN), and ϵ-greedy strategy. In Section 3, a stabilizabillity condition is derived and an algorithm based on DQN is provided. An illustrative example is employed to show the effectiveness of our results, as shown in Section 4, which is followed by a brief conclusion in Section 5.

## 2 Methods

For the convenience of statement, some symbol explanations are provided first.

**Notation:** ℝ expresses the set of all real numbers. ℤ^+^ stands for the set of all positive integers. ${\mathbb{Z}}_{a,b}^{+}$ denotes the set {*a, a*+1, ⋯ , *b*}, where *a, b* ∈ ℤ^+^, *a* ≤ *b*. |*A*| is the cardinality of set *A*.

### 2.1 Probabilistic finite state machine

A PFSM is a five-tuple


(1) $$\begin{array}{l} \Lambda =\left(X,U,P,f,X_{0}\right), \end{array}$$

where the set $X:=\left\{X_{1},X_{2},\cdots \;,X_{n}\right\}$ represents a finite set of states, and $X_{0}\in X$ is the initial state. $U:=\left\{U_{1},U_{2},\cdots \;,U_{m}\right\}$ denotes a finite set of events. P:X×U×X→[0,1] is a transition probability function, and $P\left(X_{i},U_{k},X_{j}\right):=P_{X_{i},X_{j}}^{U_{k}}$ expresses the probability of PFSM (1), transiting from state $X_{i}\in X$ to state $X_{j}\in X$ under the input event $U_{k}\in U$, satisfying


$$\begin{array}{l} \underset{X_{j}\in X}{\sum }P_{X_{i},X_{j}}^{U_{k}}=1 \end{array}$$

or


$$\begin{array}{l} \underset{X_{j}\in X}{\sum }P_{X_{i},X_{j}}^{U_{k}}=0. \end{array}$$

The state transition function $f:X\times U\to 2^{X}$ describes that PFSM (1) may reach different states from one state under the same input event, where $2^{X}$ is the power set of X.

### 2.2 Markov decision process and optimization methods

A Markov decision process (MDP) is characterized by a quintuple


(2) $$\begin{array}{l} \Omega =\left(S,A,P,R,\gamma \right), \end{array}$$

where *S* is a set of states, *A* is a set of actions, *P* is a state transition probability function, *R* is a reward function, and γ ∈ [0, 1] is a discount factor that determines the trade-off between short-term and long-term gains.

MDP (2) may reach state *s*_*t*+1_ from state *s*_*t*_ ∈ *S* under the chosen action *a*_*t*_ ∈ *A*, and its probability is determined by the function $P_{s_{t},s_{t+1}}^{a_{t}}=P\left(s_{t+1}|s_{t},a_{t}\right)$. The expected one-step reward from state *s*_*t*_ to state *s*_*t*+1_ via action *a*_*t*_ is as follows:


$$\begin{array}{l} R_{s_{t},s_{t+1}}^{a_{t}}=𝔼\left[r_{t+1}|s_{t},a_{t}\right] \end{array}$$

where *r*_*t*+1_ = *r*_*t*+1_(*s*_*t*_, *a*_*t*_, *s*_*t*+1_) represents the immediate return after adopting action *a*_*t*_ at time *t*, and 𝔼[·] is the expected value of [·].

The objective of MDP (2) is to determine an optimal policy π. This policy can maximize the expected return 𝔼_π_[*G*_*t*_] under policy π where


$$\begin{array}{l} G_{t}=\overset{\infty }{\underset{k=0}{\sum }}\gamma^{k}r_{t+k+1}. \end{array}$$

For a given policy π, the value function of a state *s*_*t*_, denoted by *v*_π_(*s*_*t*_), is the expected return of MDP (2) taking an action according to the policy π at time step *t*:


(3) $$\begin{array}{l} v_{\pi }\left(s_{t}\right)={𝔼}_{\pi }\left[\overset{\infty }{\underset{k=0}{\sum }}\gamma^{k}r_{t+k+1}|s_{t}\right],\forall s_{t}\in S. \end{array}$$

The optimal policy is as follows:


(4) $$\begin{array}{l} \pi^{*}\left(s_{t},a_{t}\right)=\underset{\pi \in \Pi }{\arg\max}v_{\pi }\left(s_{t}\right),\forall s_{t}\in S \end{array}$$

where Π is the set of all admissible policies.

From (4), it is easy to understand $v^{*}\left(s_{t}\right)=v_{\pi^{*}}\left(s_{t}\right)$. Since *v*_π_(·) satisfies the Bellman equation, we have


(5) $$\begin{array}{l} v^{*}\left(s_{t}\right)=\underset{a\in A}{\max}\underset{s\in S}{\sum }P_{s_{t},s}^{a}\left[R_{s_{t},s}^{a}+\gamma v^{*}\left(s\right)\right] \end{array}$$

Similarly, the action-value function describes the cumulative return from state-action (*s*_*t*_, *a*_*t*_) under policy π


(6) $$\begin{array}{l} q_{\pi }\left(s_{t},a_{t}\right)={𝔼}_{\pi }\left[\overset{\infty }{\underset{k=0}{\sum }}\gamma^{k}r_{t+k+1}|s=s_{t},a=a_{t}\right],\forall a_{t}\in A. \end{array}$$

By substituting (3) into (6), we can obtain


$$\begin{array}{l} q_{\pi }\left(s_{t},a_{t}\right)={𝔼}_{\pi }\left[r_{t+1}+\gamma v_{\pi }\left(s_{t+1}\right)\right], \end{array}$$

which represents the expected return of action *a*_*t*_ adopted by MDP (2) at state *s*_*t*_, following policy π. The action-value function under optimal strategy π^*^ is called as the optimal action-value function,i.e., $q^{*}\left(s_{t},u_{t}\right):=q_{\pi^{*}}\left(s_{t},a_{t}\right),\forall s_{t}\in S,\forall a_{t}\in A$. Since $v^{*}\left(s_{t}\right)=\underset{a}{\max}q^{*}\left(s_{t},a\right)$, from (5), we can get


$$\begin{array}{l} q^{*}\left(s_{t},a_{t}\right)=\underset{s\in S}{\sum }P_{s_{t},s}^{a_{t}}\left[R_{s_{t},s}^{a_{t}}+\gamma \underset{a}{\max}q^{*}\left(s,a\right)\right]. \end{array}$$

Therefore, if MDP (2) exists an optimal deterministic policy, it can be expressed as follows:


$$\begin{array}{l} \mu^{*}\left(s_{t}\right)=\underset{a\in A}{\arg\max}q^{*}\left(s_{t},a\right),\forall s_{t}\in S. \end{array}$$

DQN is such a technique that combines Q leaning with arificial neural networks (ANNs), providing an effective approach to decision-making problems in dynamic and uncertain environments. It uses ANNs to construct parametric models and estimate action value functions online. Compared with Q learning, the main advantages of DQN are as follows: (1) DQN uses ANNs to approximate Q functions, overcoming the issue of limited capacity in Q tables and enabling the algorithm to handle high-dimensional state spaces. (2) DQN makes full use of empirical knowledge.

Q learning updates the value function according to the following temporal difference (TD) formula:


(7) $$\begin{array}{l} q\left(s_{t},a_{t}\right)\leftarrow q\left(s_{t},a_{t}\right)+\alpha \left[r_{t+1}+\gamma \underset{a^{\prime }}{\max}q\left(s_{t+1},a^{\prime }\right)-q\left(s_{t},a_{t}\right)\right], \end{array}$$

where $r_{t+1}+\gamma \max_{a^{\prime }}q\left(s_{t+1},a^{{\;}^{\prime }}\right)$ is the TD target, $r_{t+1}+\gamma \underset{a^{{\;}^{\prime }}}{\max}q\left(s_{t+1},a^{{\;}^{\prime }}\right)-q\left(s_{t},a_{t}\right)$ is the TD error δ, and 0 < α ≤ 1 is a constant that determines how quickly the past experiences are forgotten.

When dealing with high-dimensional complex systems, the action-value function *q*(*s, a*), as described in Equation (7), is approximated by an ANN to reduce computational complexity. This can be achieved by minimizing the following loss function


(8) $$\begin{array}{l} L\left(\theta_{t}\right)={\left(r_{t+1}+\gamma \underset{a^{\prime }}{\max}q\left(s_{t+1},a^{\prime };\theta_{t}^{-}\right)-q\left(s_{t},a_{t};\theta_{t}\right)\right)}^{2}, \end{array}$$

where the parameter $\theta_{t}^{-}$ is a periodic copy of the current network parameter θ_*t*_.

By differentiating Equation (8), we have


(9) $$\begin{array}{r} {▽}_{\theta_{t}}ℒ(\theta_{t})=2(r_{t+1}+\gamma \underset{a^{\prime }}{\max}q(s_{t+1},a^{\prime };\theta_{t}^{-})\\ -q(s_{t},a_{t};\theta_{t}))(-{▽}_{\theta_{t}}q(s_{t},a_{t};\theta_{t})), \end{array}$$

where ▽_θ_*t*__*q*(*s*_*t*_, *a*_*t*_; θ_*t*_) represents the gradient of *q*(*s*_*t*_, *a*_*t*_; θ_*t*_) with respect to the parameter θ_*t*_.

We choose the gradient descent method as the optimization strategy


(10) $$\begin{array}{l} \theta_{t+1}=\theta_{t}-\frac{\alpha }{2}{▽}_{\theta_{t}}L\left(\theta_{t}\right). \end{array}$$

By substituting Equations (9) into (10), we obtain an update formula for parameter θ_*t*_


$$\begin{array}{r} \theta_{t+1}=\theta_{t}+\alpha [r_{t+1}+\gamma \underset{a^{\prime }}{\max}q(s_{t+1},a^{\prime };\theta_{t}^{-})\\ -q(s_{t},a_{t};\theta_{t})]{▽}_{\theta_{t}}q(s_{t},a_{t};\theta_{t}). \end{array}$$

Finally, the ϵ-greedy strategy is used for action selection. Specifically, an action is chosen randomly with probability ϵ ∈ ℝ(0 < ϵ ≤ 1), and the best estimated action is chosen with probability 1−ϵ. As learning progresses, ϵ gradually decreases, and the policy is shifted from exploring the action space to exploiting the learned Q values. The policy π(*a*∣*s*) is as follows:


π(a∣s)={1−ϵ+ϵ|A| if a=arg maxa∈Aq(s,a)ϵ|A| other actions ,

where π(*a*∣*s*) is the probability of MDP (2) selecting action *a* at state *s*. $\underset{a\in A}{\arg\max}q\left(s,a\right)$ stands for the action with the highest estimated Q value for state *s*.

## 3 Results

We first give a definition.

**Definition 1**: Assume that $X_{e}$ is an equilibrium state of PFSM (1). The PFSM is said to be feedback stabilizable to $X_{e}$ with probability one, if for any initial state $X_{i}\in X$, there exists a control sequence $\text{U}:=U_{l_{1}},U_{l_{2}},\cdots \;,U_{l_{k}}\in U$, such that $P_{X_{i},X_{e}}^{\text{U}}=1$.

We define an attraction domain ${ℜ}_{k}\left(X_{e}\right)$ for an equilibrium state $X_{e}$, which is a set of states that can reach $X_{e}$ in *k* steps.


(11) $$\begin{array}{r} {ℜ}_{k}(X_{e})=\{X_{i}\in X|\text{there exists a control sequence }U:\\ =U_{l_{1}},U_{l_{2}},\cdots ,U_{l_{k}}\in U,\text{ such that }P_{X_{i},X_{e}}^{U}=1\}. \end{array}$$

Next, we give an important result.

**Theorem 1:** Assume that $X_{e}$ is an equilibrium state of PFSM (1). The PFSM is feedback stabilizable to $X_{e}$ with probability one, if and only if there exists an integer ρ ≤ *n*−1 such that


(12) $$\begin{array}{l} {ℜ}_{\rho }\left(X_{e}\right)=X. \end{array}$$

**Proof** (Necessity): Assume that PFSM (1) is feedback stabilizable to the equilibrium state $X_{e}$ with probability one. Then, according to Definition 1, for any initial state $X_{i}$, there exists a control sequence $\text{U}:=U_{l_{1}},U_{l_{2}},\cdots \;,U_{l_{\rho }}$, such that $P_{X_{i},X_{e}}^{\text{U}}=1$, namely $X_{i}\in {ℜ}_{k}\left(X_{e}\right)$. Due to the fact that the state space is a finite set, there must be an integer ρ, such that ${ℜ}_{\rho }\left(X_{e}\right)=X$ holds.

(Sufficiency): Assume that Equation (12) holds. For any initial state $X_{i}\in X$, we have $X_{i}\in {ℜ}_{\rho }\left(X_{e}\right)$. From Equation (11), there exists a positive integer ρ and a control sequence $\text{U}:=U_{l_{1}},U_{l_{2}},\cdots \;,U_{l_{\rho }}$ such that $X_{i}$ can be driven to $X_{e}$ by **U** in ρ steps with probability one. According to Definition 1, PFSM (1) is feedback stabilizable to $X_{e}$ with probability one.      ■

We cast the feedback control problem of PFSM (1) into a model-free reinforcement learning framework. The main aim is to find a state feedback controller, which can guarantee the finite time stabilization of PFSM (1). This means that all states can be controlled and brought to an equilibrium state within finite steps. Therefore, PFSM (1) is rewritten as (X,U,P,R,γ), where *P* is unknown. The stabilization problem of PFSM (1) is formulated as follows:


(13) $$\begin{array}{l} \begin{array}{cc} \underset{\mu \left(\cdot \right)}{\max}{𝔼}_{\mu } & \left[\overset{\infty }{\underset{t=0}{\sum }}\gamma^{t}r_{t+1}\left(X_{t},U_{t},X_{t+1}\right)\right],\forall X_{0}\in X \end{array} \end{array}$$

subject to (1),

where


$$r_{t+1}=\{\begin{array}{l} 1,\text{if }X_{t+1}=X_{e}\\ -0.1,\text{otherwise}. \end{array}$$

The objective of Equation (13) is to find an action **U** that maximizes the action-value function q^*^ among all possible actions in U. Therefore, for any state $X_{t}$ and external condition $X_{e}$, the optimal state feedback control law of PFSM (1) is as follows:


$$\begin{array}{l} \mu^{*}\left(X_{t},X_{e}\right)=\underset{\text{U}\in U}{\arg\max}q^{*}\left(X_{t},\text{U},X_{e};\theta^{-}\right),\forall X_{t}\in X. \end{array}$$

**Figure 1:**
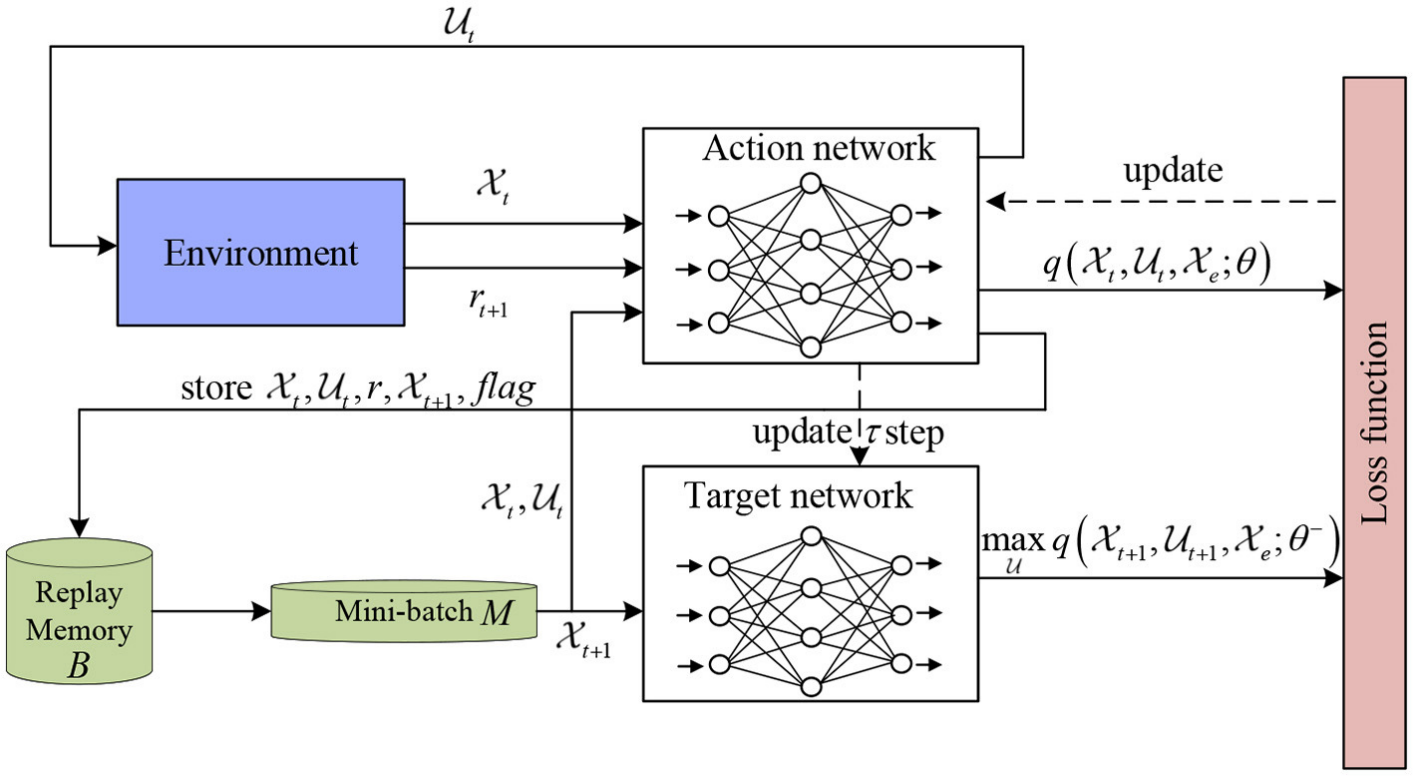
Structure diagram of DQN.

Based on the above discussion, we are ready to introduce an algorithm to design an optimal feedback controller (see Algorithm 1). It should be noted that in this algorithm, DQN uses two ANNs. The structure diagram of DQN is shown in Figure 1.

**Algorithm 1:**
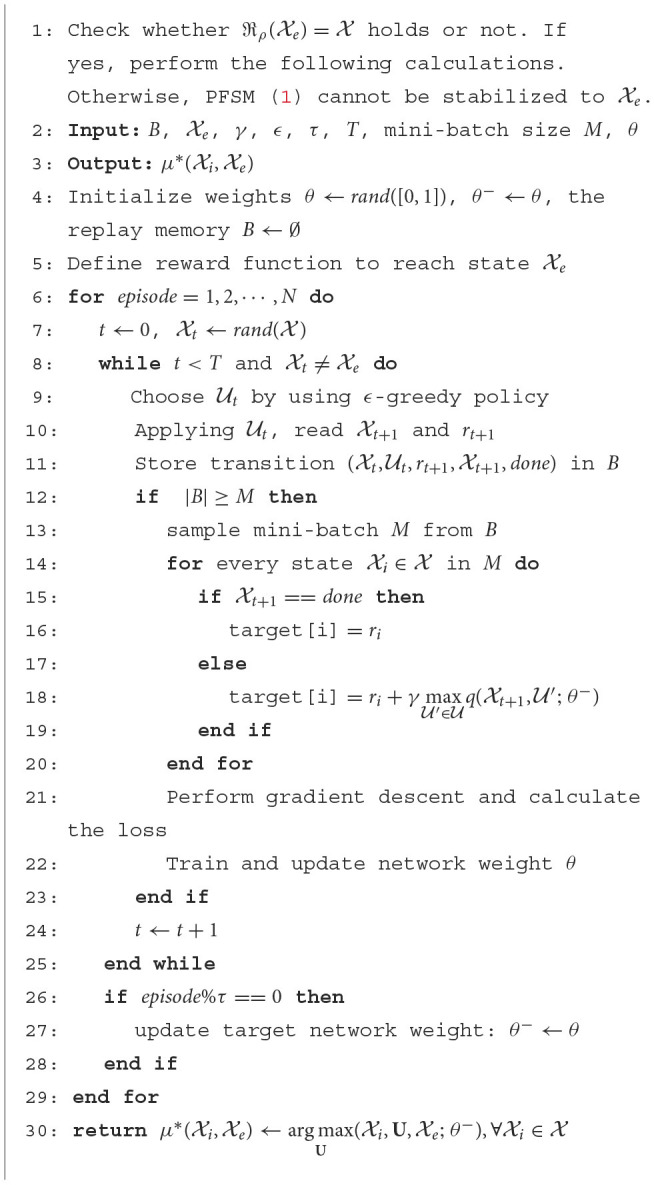
State feedback stabilization of PFSM (1) based on deep Q-network.

**Remark 1:** This algorithm is mainly used to solve the stabilization problem of high dimensional PFSMs. For small or micro-scale PFSMs, it is slightly more complex. In this case, we can choose the STP method. Therefore, Algorithm 1 and the STP method complement each other.

According to the results calculated by Algorithm 1, a state feedback controller can be given. Specifically, from Algorithm 1, the result is an optimal policy. Assume that $\mu^{*}\left(X_{i},X_{e}\right)$ is the calculation result. Then, we get a state feedback controller $\mu_{i}^{*}:=\mu^{*}\left(X_{i},X_{e}\right)$, $\forall i\in {\mathbb{Z}}_{1,n}^{+}$.

## 4 Discussion

**Example 1:** Consider a PFSM


(14) $$\begin{array}{l} X_{1}(t+1)=\{\begin{array}{l} f(X_{1}(t),U_{1})=\{\begin{array}{l} X_{2},P=0.5\\ X_{5},P=0.5 \end{array}\\ f(X_{1}(t),U_{2})=X_{1},P=1.0\\ f(X_{1}(t),U_{3})=\{\begin{array}{l} X_{1},P=0.6\\ X_{3},P=0.4, \end{array} \end{array}\;X_{2}(t+1)=\{\begin{array}{l} f(X_{2}(t),U_{1})=X_{2},P=1.0\\ f(X_{2}(t),U_{2})=\{\begin{array}{l} X_{3},P=0.7\\ X_{6},P=0.3 \end{array}\\ f(X_{2}(t),U_{3})=\{\begin{array}{l} X_{2},P=0.5\\ X_{4},P=0.5, \end{array} \end{array}\\ X_{3}(t+1)=\{\begin{array}{l} f(X_{3}(t),U_{1})=X_{3},P=1.0\\ f(X_{3}(t),U_{2})=X_{3},P=1.0\\ f(X_{3}(t),U_{3})=X_{3},P=1.0, \end{array}\;X_{4}(t+1)=\{\begin{array}{l} f(X_{4}(t),U_{1})=\{\begin{array}{l} X_{4},P=0.4\\ X_{7},P=0.6 \end{array}\\ f(X_{4}(t),U_{2})=X_{5},P=1.0\\ f(X_{4}(t),U_{3})=\{\begin{array}{l} X_{4},P=0.3\\ X_{6},P=0.7, \end{array} \end{array}\\ X_{5}(t+1)=\{\begin{array}{l} f(X_{5}(t),U_{1})=X_{5},P=1.0\\ f(X_{5}(t),U_{2})=\{\begin{array}{l} X_{3},P=0.9\\ X_{6},P=0.1 \end{array}\\ f(X_{5}(t),U_{3})=\{\begin{array}{l} X_{5},P=0.8\\ X_{9},P=0.2, \end{array} \end{array}\;X_{6}(t+1)=\{\begin{array}{l} f(X_{6}(t),U_{1})=X_{3},P=1.0\\ f(X_{6}(t),U_{2})=X_{6},P=1.0\\ f(X_{6}(t),U_{3})=\{\begin{array}{l} X_{6},P=0.7\\ X_{7},P=0.3, \end{array} \end{array}\\ X_{7}(t+1)=\{\begin{array}{l} f(X_{7}(t),U_{1})=X_{7},P=1.0\\ f(X_{7}(t),U_{2})=X_{5},P=1.0\\ f(X_{7}(t),U_{3})=\{\begin{array}{l} X_{7},P=0.6\\ X_{8},P=0.4, \end{array} \end{array}\;X_{8}(t+1)=\{\begin{array}{l} f(X_{8}(t),U_{1})=\{\begin{array}{l} X_{7},P=0.7\\ X_{8},P=0.3 \end{array}\\ f(X_{8}(t),U_{2})=X_{9},P=1.0\\ f(X_{8}(t),U_{3})=\{\begin{array}{l} X_{8},P=0.9\\ X_{1},P=0.1, \end{array} \end{array}\\ X_{9}(t+1)=\{\begin{array}{l} f(X_{9}(t),U_{1})=X_{6},P=1.0\\ f(X_{9}(t),U_{2})=X_{9},P=1.0\\ f(X_{9}(t),U_{3})=\{\begin{array}{l} X_{9},P=0.5\\ X_{2},P=0.5, \end{array} \end{array}\;X_{10}(t+1)=\{\begin{array}{l} f(X_{10}(t),U_{1})=\{\begin{array}{l} X_{11},P=0.3\\ X_{12},P=0.7 \end{array}\\ f(X_{10}(t),U_{2})=X_{10},P=1.0\\ f(X_{10}(t),U_{3})=\{\begin{array}{l} X_{10},P=0.4\\ X_{13},P=0.6, \end{array} \end{array}\\ X_{11}(t+1)=\{\begin{array}{l} f(X_{11}(t),U_{1})=X_{11},P=1.0\\ f(X_{11}(t),U_{2})=\{\begin{array}{l} X_{12},P=0.5\\ X_{14},P=0.5 \end{array}\\ f(X_{11}(t),U_{3})=\{\begin{array}{l} X_{11},P=0.7\\ X_{15},P=0.3, \end{array} \end{array}\;X_{12}(t+1)=\{\begin{array}{l} f(X_{12}(t),U_{1})=\{\begin{array}{l} X_{16},P=0.6\\ X_{17},P=0.4 \end{array}\\ f(X_{12}(t),U_{2})=X_{12},P=1.0\\ f(X_{12}(t),U_{3})=\{\begin{array}{l} X_{12},P=0.8\\ X_{18},P=0.2, \end{array} \end{array}\\ X_{13}(t+1)=\{\begin{array}{l} f(X_{13}(t),U_{1})=X_{13},P=1.0\\ f(X_{13}(t),U_{2})=\{\begin{array}{l} X_{14},P=0.9\\ X_{19},P=0.1 \end{array}\\ f(X_{13}(t),U_{3})=\{\begin{array}{l} X_{13},P=0.5\\ X_{20},P=0.5, \end{array} \end{array}\;X_{14}(t+1)=\{\begin{array}{l} f(X_{14}(t),U_{1})=\{\begin{array}{l} X_{15},P=0.8\\ X_{16},P=0.2 \end{array}\\ f(X_{14}(t),U_{2})=X_{14},P=1.0\\ f(X_{14}(t),U_{3})=\{\begin{array}{l} X_{17},P=0.6\\ X_{18},P=0.4, \end{array} \end{array}\\ X_{15}(t+1)=\{\begin{array}{l} f(X_{15}(t),U_{1})=X_{15},P=1.0\\ f(X_{15}(t),U_{2})=\{\begin{array}{l} X_{16},P=0.7\\ X_{20},P=0.3 \end{array}\\ f(X_{15}(t),U_{3})=\{\begin{array}{l} X_{19},P=0.5\\ X_{15},P=0.5, \end{array} \end{array}\;X_{16}(t+1)=\{\begin{array}{l} f(X_{16}(t),U_{1})=X_{16},P=1.0\\ f(X_{16}(t),U_{2})=\{\begin{array}{l} X_{17},P=0.8\\ X_{18},P=0.2 \end{array}\\ f(X_{16}(t),U_{3})=\{\begin{array}{l} X_{16},P=0.6\\ X_{19},P=0.4, \end{array} \end{array}\\ X_{17}(t+1)=\{\begin{array}{l} f(X_{17}(t),U_{1})=X_{17},P=1.0\\ f(X_{17}(t),U_{2})=\{\begin{array}{l} X_{18},P=0.9\\ X_{20},P=0.1 \end{array}\\ f(X_{17}(t),U_{3})=\{\begin{array}{l} X_{17},P=0.7\\ X_{19},P=0.3, \end{array} \end{array}\;X_{18}(t+1)=\{\begin{array}{l} f(X_{18}(t),U_{1})=X_{18},P=1.0\\ f(X_{18}(t),U_{2})=\{\begin{array}{l} X_{19},P=0.8\\ X_{20},P=0.2 \end{array}\\ f(X_{18}(t),U_{3})=\{\begin{array}{l} X_{18},P=0.5\\ X_{1},P=0.5, \end{array} \end{array}\\ X_{19}(t+1)=\{\begin{array}{l} f(X_{19}(t),U_{1})=X_{19},P=1.0\\ f(X_{19}(t),U_{2})=\{\begin{array}{l} X_{20},P=0.6\\ X_{1},P=0.4 \end{array}\\ f(X_{19}(t),U_{3})=\{\begin{array}{l} X_{19},P=0.8\\ X_{2},P=0.2, \end{array} \end{array}\;X_{20}(t+1)=\{\begin{array}{l} f(X_{20}(t),U_{1})=X_{20},P=1.0\\ f(X_{20}(t),U_{2})=\{\begin{array}{l} X_{1},P=0.7\\ X_{3},P=0.3 \end{array}\\ f(X_{20}(t),U_{3})=\{\begin{array}{l} X_{20},P=0.9\\ X_{4},P=0.1, \end{array} \end{array} \end{array}$$

where $X_{i}\left(t\right)$ represents the *i*-th state of PFSM (14) at time step *t*. It is easy to observe that $X_{3}$ is an equilibrium state.

We now use Algorithm 1 to compute a state feedback controller to stabilize PFSM (14) to $X_{3}$. The computation is performed on a computer with Intel i5-11300H processor, 2.6 GHz frequency, 16 GB RAM, and Python 3.7 software. We adopt TensorFlow in Keras to train the DQN model, where the discount factor γ is 0.99, the rang for ϵ in ϵ-greedy policy is from 0.05 to 1.0, and the sizes of memory buffer *B* and mini-batch *M* are 10,000 and 128, respectively.

Through calculation, we obtain a state feedback controller


(15) $$\begin{array}{l} \mu_{i}^{*}=\mu^{*}\left(X_{i},X_{3}\right),i\in \left[1,20\right], \end{array}$$

which is shown in Table 1.

**Table 1:** A state feedback controller of PFSM (14).

State	$X_{1}$	$X_{2}$	$X_{3}$	$X_{4}$	$X_{5}$	$X_{6}$	$X_{7}$	$X_{8}$	$X_{9}$	$X_{10}$	$X_{11}$	$X_{12}$	$X_{13}$	$X_{14}$	$X_{15}$	$X_{16}$	$X_{17}$	$X_{18}$	$X_{19}$	$X_{20}$
Action	$U_{1}$	$U_{2}$	$U_{1}$	$U_{1}$	$U_{2}$	$U_{1}$	$U_{2}$	$U_{2}$	$U_{1}$	$U_{3}$	$U_{2}$	$U_{3}$	$U_{3}$	$U_{3}$	$U_{2}$	$U_{2}$	$U_{2}$	$U_{3}$	$U_{2}$	$U_{2}$

**Figure 2:**
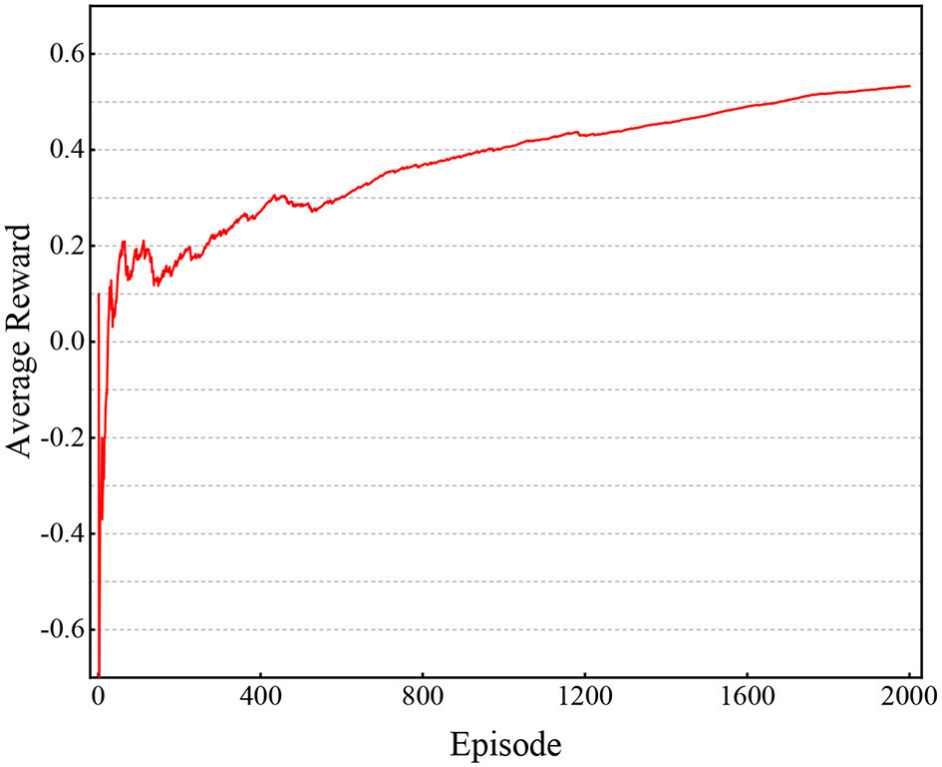
Performance of Algorithm 1 in Example 1.

Model (14) is a PFSM with 20 states, which is not a simple system. Here, we utilize average rewards to track the performance during training (see Figure 2). It is easy to observe that as training time goes on, the performance inceases and tends to be stable. We put the state feedback controller (15), as shown in Table 1, into PFSM (14) and get a closed-loop system.


(16) $$\begin{array}{l} X_{1}(t+1)=\{\begin{array}{l} X_{2},P=0.5\\ X_{5},P=0.5, \end{array}\;X_{2}(t+1)=\{\begin{array}{l} X_{3},P=0.7\\ X_{6},P=0.3, \end{array}\\ X_{3}(t+1)=X_{3},P=1.0,\;X_{4}(t+1)=\{\begin{array}{l} X_{4},P=0.4\\ X_{7},P=0.6, \end{array}\\ X_{5}(t+1)=\{\begin{array}{l} X_{3},P=0.9\\ X_{6},P=0.1, \end{array}\;X_{6}(t+1)=X_{3},P=1.0,\\ X_{7}(t+1)=X_{5},P=1.0,\;X_{8}(t+1)=X_{9},P=1.0,\\ X_{9}(t+1)=X_{6},P=1.0,\;X_{10}(t+1)=\{\begin{array}{l} X_{10},P=0.4\\ X_{13},P=0.6, \end{array}\\ X_{11}(t+1)=\{\begin{array}{l} X_{12},P=0.5\\ X_{14},P=0.5, \end{array}\;X_{12}(t+1)=\{\begin{array}{l} X_{12},P=0.8\\ X_{18},P=0.2, \end{array}\\ X_{13}(t+1)=\{\begin{array}{l} X_{13},P=0.5\\ X_{20},P=0.5, \end{array}\;X_{14}(t+1)=\{\begin{array}{l} X_{17},P=0.6\\ X_{18},P=0.4, \end{array}\\ X_{15}(t+1)=\{\begin{array}{l} X_{16},P=0.7\\ X_{20},P=0.3, \end{array}\;X_{16}(t+1)=\{\begin{array}{l} X_{17},P=0.8\\ X_{18},P=0.2, \end{array}\\ X_{17}(t+1)=\{\begin{array}{l} X_{18},P=0.9\\ X_{20},P=0.1, \end{array}\;X_{18}(t+1)=\{\begin{array}{l} X_{18},P=0.5\\ X_{1},P=0.5, \end{array}\\ X_{19}(t+1)=\{\begin{array}{l} X_{20},P=0.6\\ X_{1},P=0.4, \end{array}\;X_{20}(t+1)=\{\begin{array}{l} X_{1},P=0.7\\ X_{3},P=0.3. \end{array} \end{array}$$

**Figure 3:**
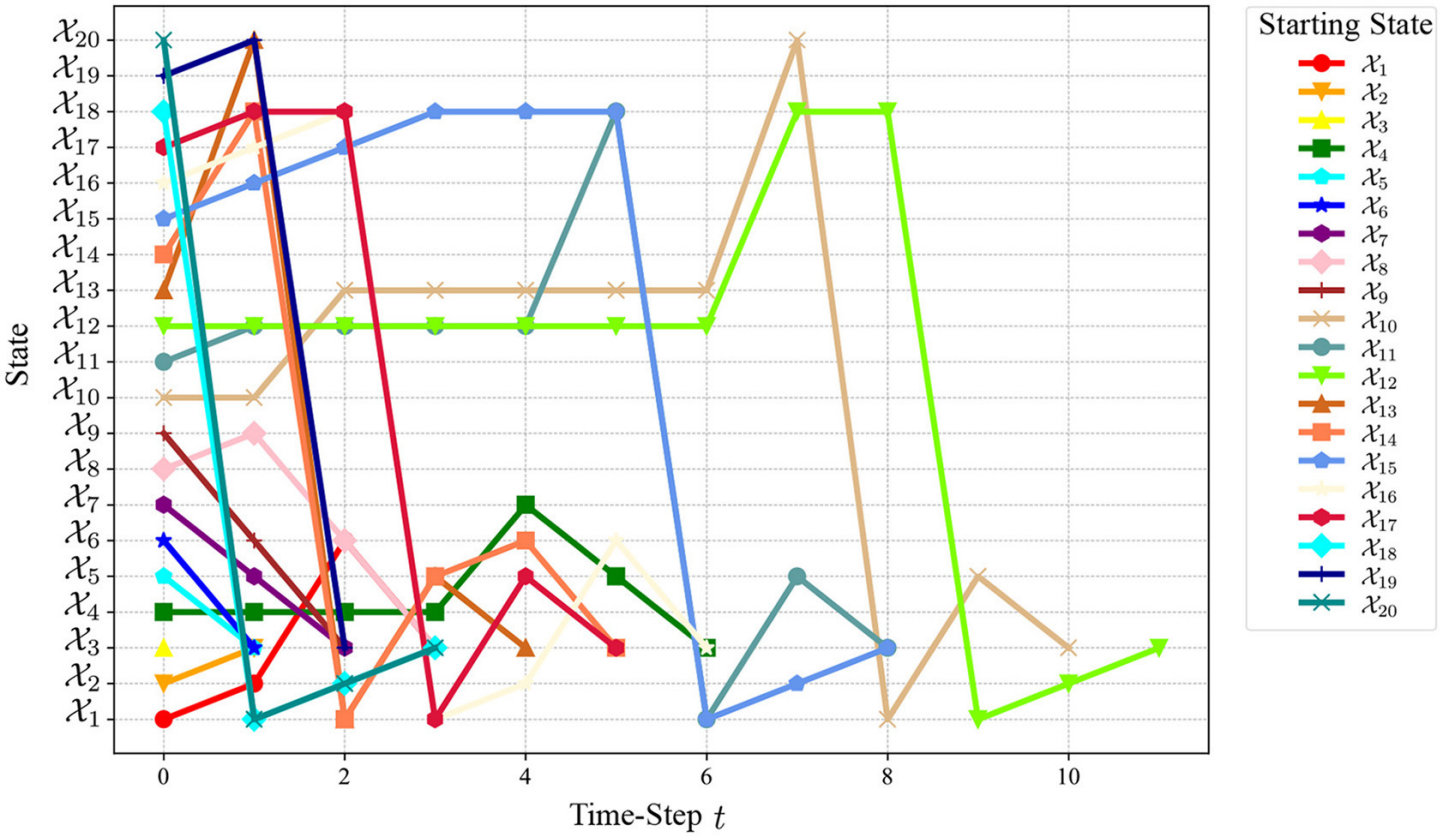
Evolution of the closed-loop system (16).

**Figure 4:**
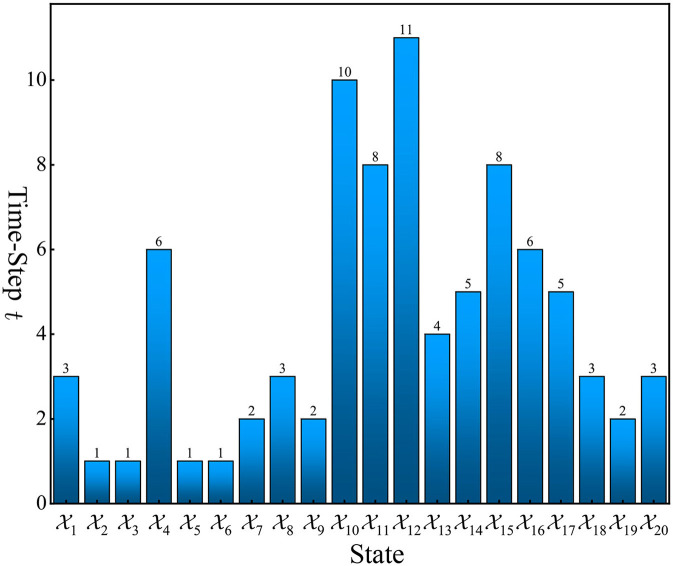
The number of steps required to stabilize PFSM (14) to $X_{3}$.

The state transition trajectory of the closed-loop system (16) starting from any initial state is shown in Figure 3. It can be observed from Figure 3 that all states reach $X_{3}$ after a finite number of steps and then stay at $X_{3}$ forever with probability one. This demonstrates the effectiveness of our controller. The number of steps required to reach $X_{3}$ for each state is shown in Figure 4. From these results, we can observe that based on DQN, Algorithm 1 can solve the stabilization problem of non-small-scale PFSMs.

## 5 Conclusion

This article studied the state feedback stabilization of PFSMs using the DQN method. The feedback stabilization problem of PFSMs was first transformed into an optimization problem. A DQN was built, whose two key parts: TD target and Q function, are approximated through neural networks. Then, based on the DQN and a stabilizability condition derived in this paper, an algorithm was developed. The algorithm can be used to calculate the optimization problem mentioned above and then solves the feedback stability problem of PFSMs. Since DQN avoids the limited capacity problem of Q learning, our algortithm can handle high-dimensional complex systems. Finally, an illustrative example is provided to show the effectiveness of our method.

## Data availability statement

The original contributions presented in the study are included in the article/supplementary material, further inquiries can be directed to the corresponding author.

## Author contributions

HT: Conceptualization, Formal analysis, Funding acquisition, Methodology, Project administration, Supervision, Writing—review & editing. XS: Conceptualization, Formal analysis, Investigation, Methodology, Validation, Writing—original draft. YH: Formal analysis, Investigation, Writing—review & editing.
